# Supplementary material for: Transcriptomic Insights into Mechanisms of Early Seed Maturation in the Garden Pea (Pisum sativum L.)
Source: Cells. 2020 Mar 23;9(3):779. doi: 10.3390/cells9030779 (PMC7140803; doi:10.3390/cells9030779)

Sprint−2,10(1)

Global Summary

%DE = 0.21

genes with fdr < 0.2 = 452 (228 + / 224 −)

genes with fdr < 0.1 = 420 (211 + / 209 −)

genes with fdr < 0.05 = 389 (197 + / 192 −)

genes with fdr < 0.01 = 339 (179 + / 160 −)

genes in genesets = 0

<FC> = 0

<t−score> = 0

<p−value> = 0.01

<fdr> = 0.79

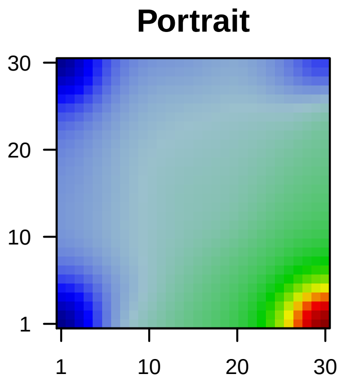

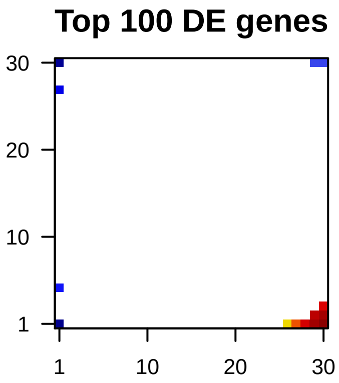

Global Genelist

Rank	ID	log(FC)	p−value	fdr	Metagene	Description
1	TRINITY_DN1	1.23	2e−16	2e−15	30 x 1	
2	TRINITY_DN3	1.45	2e−16	2e−15	30 x 1	
3	TRINITY_DN9	−0.67	2e−16	2e−15	30 x 30	
4	TRINITY_DN7	3.84	2e−16	2e−15	30 x 1	
5	TRINITY_DN9	0.95	2e−16	2e−15	27 x 1	
6	TRINITY_DN5	−1.08	2e−16	2e−15	30 x 30	
7	TRINITY_DN5	−1.81	2e−16	2e−15	1 x 1	
8	TRINITY_DN5	−0.88	2e−16	2e−15	1 x 30	
9	TRINITY_DN7	−1.29	2e−16	2e−15	1 x 1	
10	TRINITY_DN9	3.91	2e−16	2e−15	30 x 1	
11	TRINITY_DN9	1.18	2e−16	2e−15	30 x 1	
12	TRINITY_DN9	−0.8	2e−16	2e−15	1 x 1	
13	TRINITY_DN9	1.31	2e−16	2e−15	30 x 1	
14	TRINITY_DN2	−0.73	2e−16	2e−15	1 x 1	
15	TRINITY_DN2	0.91	2e−16	2e−15	30 x 1	
16	TRINITY_DN2	1.43	2e−16	2e−15	30 x 1	
17	TRINITY_DN2	−0.73	2e−16	2e−15	1 x 30	
18	TRINITY_DN2	−0.68	2e−16	2e−15	1 x 27	
19	TRINITY_DN2	−1.08	2e−16	2e−15	1 x 30	
20	TRINITY_DN2	−0.99	2e−16	2e−15	1 x 1	

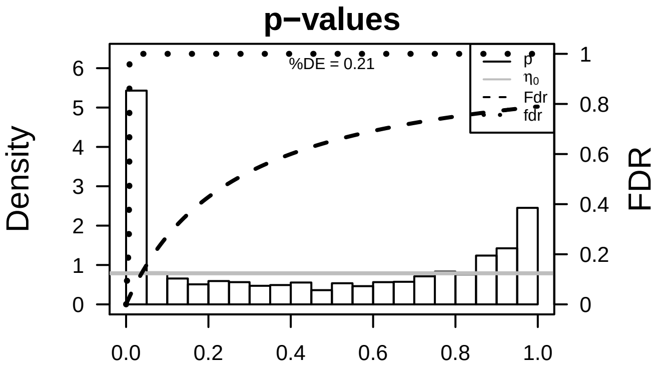

Supplement: Supplementary file 1 [file cells-09-00779-s001.zip › Supplementary materials/FileS4.zip!/FileS3/TFs - Results/Summary Sheets - Samples/Sprint.2.10.1..pdf]
